# Authentication and Key Establishment in Dynamic Wireless Sensor Networks

**DOI:** 10.3390/s100403718

**Published:** 2010-04-13

**Authors:** Ying Qiu, Jianying Zhou, Joonsang Baek, Javier Lopez

**Affiliations:** 1 Cryptography and Security Department, Institute for Infocomm Research, 138632, Singapore; E-Mails: jyzhou@i2r.a-star.edu.sg (J.Z.); jsbaek@i2r.a-star.edu.sg (J.B.); 2 Department of Computer Science, University of Malaga, 29071 Malaga, Spain; E-Mail: jlm@lcc.uma.es

**Keywords:** wireless sensor networks, authentication, key management, energy efficiency, scalability

## Abstract

When a sensor node roams within a very large and distributed wireless sensor network, which consists of numerous sensor nodes, its routing path and neighborhood keep changing. In order to provide a high level of security in this environment, the moving sensor node needs to be authenticated to new neighboring nodes and a key established for secure communication. The paper proposes an efficient and scalable protocol to establish and update the authentication key in a dynamic wireless sensor network environment. The protocol guarantees that two sensor nodes share at least one key with probability 1 (100%) with less memory and energy cost, while not causing considerable communication overhead.

## 1. Introduction

The demand of wireless sensor networks (WSNs) is growing exponentially. It has turned out that the sensor networks can be widely applied in the areas of healthcare, environment monitoring, and the military. One of the surveys on WSNs points out that, in the near future, wireless sensor networks will be an integral part of our lives, more so than the present-day personal computer [1].

A sensor node has low capability in terms of power, computation, storage and communication. A wireless sensor network is composed of a large number of wireless sensor nodes and multi-hop communication is desired in WSNs. As a result, security in wireless sensor networks has six challenges to overcome: (i) the wireless nature of communication, (ii) resource limitations of sensor nodes, (iii) very large and dense WSNs, (iv) lack of fixed infrastructure, (v) unknown network topology prior to deployment, (vi) high risk of physical attacks on unattended sensors [2].

Baek *et al.* [3] conducted a survey of several authentication schemes used in wireless sensor networks. The Radio Resource Testing scheme [4] uses non-cryptographic means to share a key. The Random Key Pre-distribution scheme [5] is a popular key management scheme in WSNs, which requires small computation and communication overheads, but is not suitable for environments where node compromise is common and scalability is in high demand. Time Synchronized (μTESLA) [6], One Time Signature [7], and Public Key Authentication [8] are used for broadcast authentication. Kerberos is a network authentication system that uses a trusted third party (or trusted authority) to authenticate two entities (*i.e.*, to prove their identity to one another) by issuing a shared session key between them [9].

However, the μTESLA scheme has the problem of time synchronization and delayed authentication. One time signature schemes and public key authentication schemes in general have thus far been costly to implement over sensor nodes in terms of computational, communication and storage overheads except for some specific cryptographic applications based on elliptic curve primitives [10,11]. The messages exchanged in Kerberos can have a payload of several kilobytes, which makes the standard Kerberos protocol rather unpractical for use in WSNs where the communication is very costly due to the energy consumption during data transmission.

Zhang *et al.* [12] proposed a scheme called the pre-distribution and local collaboration-based group rekeying (PCGR). The design's ideas are: (1) Future keys can be preloaded to individual nodes before deployment to avoid the high overhead in securely disseminating new keys at the key updating time. (2) Neighbors can collaborate with each other to effectively protect and appropriately use the preloaded keys; the local collaboration also avoids the high cost of the centralized management. However, this scheme requires some strong assumptions: (1) Most of sensor nodes have the capability to detect their compromised neighbor nodes. (2) A sensor node could be assigned to multiple groups, but the nodes in the same group shared a unique key.

In recent years, the use of pairing-based cryptographic schemes in WSN environments has been proposed for stronger protection. There are some works in the literature that aim to make the paring-based schemes faster and smaller [13–16]. However, such schemes still have to address the issues of key revoking since the sensor nodes can easily be compromised. Also, from the perspective of the security layers, many wireless protocols use outdated encryption algorithms, which have proven unsuitable for hardware usage, particularly with handheld devices [17].

With the aforementioned limitations of the existing solutions in mind, we now propose a secure protocol in dynamic WSN, addressing all of the following issues:

- A moving sensor node needs to change its attached routers (or cluster heads) frequently.
- A router (or cluster head) needs to ensure a joining node is not a malicious sensor.
- A moving node needs to establish a secure tunnel with the new router (or cluster head).
- The energy consumption for establishing the secure tunnel must be minimal.

One of the important novel features of our solution is that we use cluster heads as sub-base-stations to execute key establishment. This way, the total dependency on the base station for key establishment can be avoided. Also, this approach reduces the hops between two communicating ends and hence results in reduction of the communication cost.

The remaining part of this paper is organized as follows: Section 2 describes the network assumptions. Section 3 briefs the usage of a general Random Pair-Wise Key scheme. Section 4 introduces our protocol. Section 5 is the security analysis of this scheme. The comparison and performance analysis is in Section 6. Finally, the conclusions are presented in Section 7.

## 2. Network Assumptions

In this paper, we consider a scenario in which a sensor node roams within a very large and distributed WSN, consisting of a large number of sensor nodes. It is a typical scenario that is widely adopted in hospital environments as the patients or doctors equipped with sensors roam across each department in the hospital.

**Figure 1.**
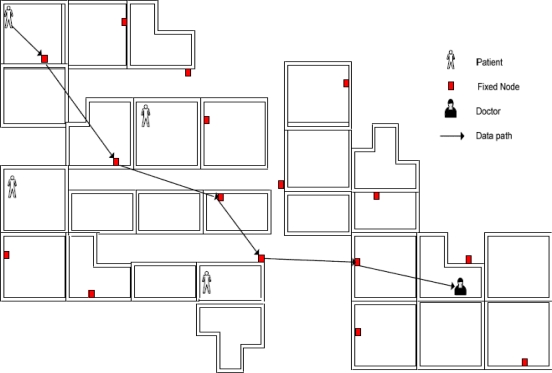
A dynamic WSN scenario in hospital application.

CodeBlue [18–20] is one of the popular sensor network architectures deployed in medical sensor network environments. Figure 1 depicts the typical CodeBlue architecture. A patient who carries the sensor nodes can move freely within the range of a hospital. When a wireless sensor node is moving, its routing path and neighborhood keep changing. The moving node needs to be authenticated to the new neighbors and to establish a key for secure communication.

The above network architecture reflects the problems described in Section 1: (a) composition by a large number of sensor nodes; (b) communication based on wireless multi-hop mechanism; (c) no fixed infrastructure; (d) the possible location change of sensor node (patient). Therefore, the challenges of this network assumption are how to establish a secure channel with these routers. It is also assumed in the solution that the base-station is always online and provides the full utilities.

## 3. Shared-Key Discovery

In the WSN environment, as data transmission consumes much more energy than computation, the probabilistic solution is widely accepted in order to reduce the storage and communication overhead during key establishment.

So far in the literature, numerous random key pre-distribution schemes have been proposed. For example, in Chan *et al.*’s scheme [21], each sensor node stores a random set of *Np* dedicated pair-wise keys to achieve the probability *p* that two nodes share a key. At the key setup phase, each node ID is matched with *Np* other randomly selected node IDs with probability *p*. A distinct pair-wise key is generated for each ID pair, and is stored in both nodes’ key-chain along with the ID of the other party. During the shared-key discovery phase, each node broadcasts its ID so that neighboring nodes can tell if they share a common pair-wise key. Note that Chan *et al.*’s scheme reduces the storage overhead by sacrificing key connectivity, but it still provides perfect key resilience.

In our scenario, we assume that *a sensor node (carried by a patient) can move within a hospital*. As each sensor’s memory is severely constrained, each sensor may only store a small set of keys randomly selected from a key pool at the deployment. Two nodes may use any existing key discovery protocol (e.g., the solution proposed in [21]) to find a common key from their own sets. If the common key is not found, our key establishment scheme will be initiated. The reason why we bind a general pre-shared key discovery phase to our protocol is to reduce the energy cost as much as possible.

## 4. Dynamic Authentication and Key Establishment Protocol

### 4.1. Basic Protocol

Due to the limited storage of sensor nodes, the pre-shared key-pair is not always available between the roaming node and its new neighbors in the circumstance of a dynamic node roaming within large WSNs (e.g., in hospitals and nuclear power plants). Therefore it requires an efficient and scalable protocol to establish and update the keys among nodes for secure communications.

**Figure 2.**
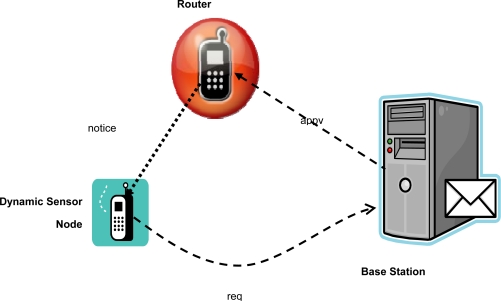
The basic architecture and message flow of our protocol.

Figure 2 shows the basic architecture and message flow of our protocol for authentication and key establishment in dynamic WSNs. When a dynamic sensor node moves to a new area and wants to attach to a router or a cluster head in this area, it first sends a request message to the base station (refer to Figure 2).

(1) req={Src=SN,Dst=BS,RT‖R0‖MAC(KBN,SN||RT||R0)}

where *Src* and *Dst* denote the source and destination address of a message respectively. *SN, BS* and *RT* are identifiers for sensor node, base station and router, respectively. *R_0_* denotes a random number generated by the sensor node. *MAC* indicates the message authentication code algorithm with a key and *K_BN_* is the shared secret key between the base station and the sensor node.

After receiving the *req* message, the base station will check its revocation list whether the sensor node has been revoked. If the sensor node is acceptable, then the base station verifies the *MAC* message. If the result is positive, the base station will generate a session key *K_NR_* for the roaming sensor node and the router (or cluster head).

(2) $$K_{\mathrm{NR}}=H(K_{\mathrm{BN}},\mathrm{SN}||R_{0}||R_{1})$$

where *H* is a keyed one-way hash function, and *R_1_* is the random number selected by the base station. The base station then sends an approval message *appv* with the session key to the router:

(3) $$\mathrm{appv}=\{\mathrm{Src}=\mathrm{BS},\mathrm{Dst}=\mathrm{RT},E(K_{\mathrm{BT}},\mathrm{SN}||R_{0}||R_{1}||K_{\mathrm{NR}})\}$$

where *E* is an encryption algorithm, and *K_BT_* is the shared secret key between the base station and the router.

After receiving the *appv* message, the router decrypts the payload and extracts the session key *K_NR_*, and then sends a notice to the sensor node.

(4) $$\mathrm{notice}=\{\mathrm{Src}=\mathrm{RT},\mathrm{Dst}=\mathrm{SN},R_{0}||R_{1}||\mathrm{MAC}(K_{\mathrm{NR}},\mathrm{RT}||\mathrm{SN}||R_{0}||R_{1})\}$$

Upon getting the notice message, the sensor node extracts the random numbers *R_0_* and *R_1_*. After checking if the received random number *R_0_* is equal to the original *R_0_*, the sensor node recalculates the session key *K_NR_ = H(K_BN_, SN*||*R_0_*||*R_1_)* and then verifies the *MAC* value. If the result is positive, the sensor node will use the session key for the communication with this router afterwards. In practice, the router could be any sensor node that the dynamic sensor node wants to connect to.

### 4.2. Key Management

In order to manage the keys, every sensor node maintains a table, called “Key Cache”. Table 1 shows the structure of the Key Cache.

When a sensor node, say node *N*, wants to connect to other sensor node, say node *R*, it executes the following procedure:

1. Checks first if there is an existing key pair between them.
2. Otherwise, processes the subroutine of shared-key discovery to find a common key between node *N* and node *R* based on those “PreSharedKeys” in their key caches.
3. If there is still no common key between them, the sensor node allocates an entry in the key cache, and assigns Node ID as *node_R_*, Key Stuff as the random number *R_0_* and Key Lifetime as *0*, as shown in Table 2.
4. Then the sensor node initiates the procedure of key establishment described in the above section. After receiving the notice message, and recalculating the session key *K_NR_*, the sensor node updates the entry’s key stuff and key lifetime accordingly.
5. When the key lifetime is expired, the dynamic sensor node should re-initiate the procedure of key establishment described in the above section.
6. When the sensor node leaves the range of the connected router, the sensor node deletes the related entry from its cache table in order to save the storage. In case there is no space for adding a new entry, it may first delete the oldest key which has expired or will expire soon.

The base station also maintains a key table (Table 3) that includes the secret keys shared with all of the sensor nodes in the network.

If a node is compromised and revoked, its field of key lifetime would be marked as negative.

### 4.3. Distribution Mode

In WSNs, the more hops between two communicating ends exist, the poorer the traffic performance becomes and the more energy consumption is required. To overcome these problems, we introduce the *distribution mode*.

The major idea of distribution mode is to deploy the cluster heads as the sub-base-stations because a cluster head is more powerful than normal sensor nodes. The distribution mode includes the following steps:

1. Each cluster head manages to establish the shared key with its neighboring cluster heads after deployment. There are several ways to do this. One could embed those keys in advance if the topology is known at deployment, or use the basic protocol described in the above sections, *via* the base station. (As this is a one-time operation, the overheads may be acceptable.)
2. Each sensor node keeps two base station identifiers (IDs): one is a real base station ID; the other is a sub-base-station (the cluster head) ID. Initially, the ID of sub-base-station is a real base station.
3. After deployment, the first round for a mobile node to establish the shared key with the nearest cluster head uses the basic protocol, too.
4. When the mobile node moves, use the basic protocol to establish the shared key with the new cluster head, *via* the sub-base-station (old cluster head) rather than the real base station.
5. After successfully establishing the keys, the sensor node updates the ID of sub-base-station with the current cluster head.
6. For security reasons, each sensor node must reset its sub-base-station ID to the real base station at a specified interval (say a few hours or days, depending on the various applications) and re-establish keys with its near cluster heads *via* the real base station. If the base station does not receive any request from a sensor node, it considers the sensor node has been compromised.

The distribution mode could provide an efficient and low energy-cost solution for the shared-key establishment. The basic protocol can provide the stronger protection since it can immediately block and revoke compromised nodes.

## 5. Security Analysis

In this proposed protocol, the session key *K_NR_* between the sensor node and the router is generated by the base station and sensor node respectively, and the session key is directly sent to the router from the base station by an encrypted packet. Hence, the session key *K_NR_* is never disclosed during transmission. The session key *K_NR_* is only known by the related peers, *i.e.*, the sensor node, the base station and the router.

Referring to equation (2), the session key *K_NR_* is generated by a keyed hash function with the shared key *K_BN_* between sensor node and base station as well as two random numbers, *R_0_* and *R_1_*, which are generated by the sensor node and base station respectively. As both *R_0_* and *R_1_* are used only one time, there are not the same session keys *K_NR_*. This property is useful to against the replication attacks.

Since the session key *K_NR_* is generated by a keyed hash function with the secret key *K_BN_* between the sensor node and the base station, the different sensor nodes will have different session keys. This feature is useful to protect sensor node privacy.

Even though an eavesdropper at the edge of the sensor node can monitor and capture the random numbers *R_0_* and *R_1_* as well as the identity of the sensor node, it is still not able to regenerate the session key *K_NR_* due to lack of the secret key *K_BN_*. Without a proper session key, the routers will not forward the packets to next nodes. This attribute could prevent camouflage and traffic attacks.

Due to the fact that no trusted connection is established between sensor node and new router before the connection between them, the proposed protocol employs a random number *R_1_* issued by the base station. The sensor node needs to recalculate the *K_NR_* first based on the *R_1_* together with *K_BN_* and *R_0_*. Then using the calculated session key *K_NR_* to verify the received session key *K_NR_* and the random number *R_1_*. If the result is positive, then the sensor node will trust that the router is authorized by the base station.

Besides the function of informing the sensor node that the new session key *K_NR_* is ready to use in the router, the *notice* message also plays an important role to check if the sensor node’s address is reachable. Without this reachability check, the sensor node may claim that it is at any location rather than its real location. It could launch redirecting attacks.

The path between the base station and the router is secure because the packet between them is encrypted with a pre-shared key *K_BT_*.

The messages from the sensor node to the base station and from the router to the sensor node are authenticated by a keyed hash function. Before accepting the inward message and making further processing, the receivers must verify the authentication. Since the cost of a hash algorithm is very small, the base station and sensor node could avoid the attacks of denial of service.

In order to achieve high efficiency and low energy cost, the protocol deploys a distribution mode which uses the cluster headers as the sub-base-stations. Due to the capability of cluster header, it is not able to recognize any compromised sensor nodes in time; the protocol requires each sensor node to reset its sub-base-station ID to the real base station regularly, and to re-establish keys with its near cluster heads *via* the real base station. This step is also useful to avoid a sensor node binding a compromised cluster head for long time.

According to the above analysis, this proposed protocol, which is simple and easy to implement, can provide relatively strong protection for sensor node networks.

## 6. Comparison and Performance Analysis

In this section, we compare our protocol with some popular or latest key establish protocols used in WSNs.

### 6.1. Simplified Kerberos Protocol

**Figure 3.**
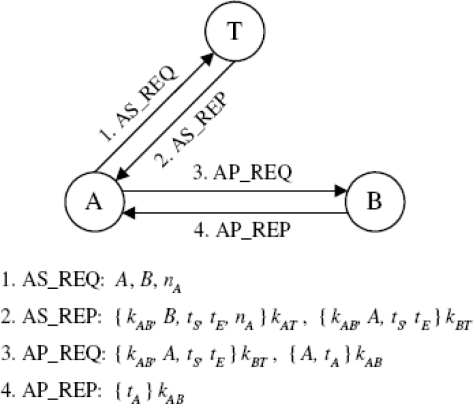
Message exchange in the simplified Kerberos protocol.

Kerberos is a network authentication system that uses a trusted third party (or trusted authority) to authenticate two entities (*i.e.*, to prove their identity to one another) by issuing a shared session key between them [9]. The messages exchanged in Kerberos can have a payload of several kilobytes, which makes the standard Kerberos protocol rather unpractical for use in sensor networks where data transfer is extremely costly in terms of energy consumption. Therefore, in early 2007, a “Simplified Kerberos Protocols” was introduced [22]. Figure 3 shows the messages exchanged in this protocol.

We assume that node identifiers (IDs) and timestamps consist of 64 bits, whereas the session key and the random nonce have the length of 128 bits and 32 bits, respectively. We also assume that the overhead of each message is 256 bits, which includes a protocol ID, a message ID, a checksum as reliable indicator of data integrity, as well as low-level (MAC and PHY) headers and footers consisting of network addresses and other bookkeeping data.

Considering the padding requirement for implementing AES encryption, the estimated message length of the Simplified Kerberos Protocol and our protocol is shown in Tables 4 and 5, respectively.

According to WINS radio module, the transmission of one bit of data requires energy of between 7.71 μJ and 10.8 μJ on the sending node, and 7.52 μJ on the receiving node, respectively. The overall energy cost for transmitting (*i.e.*, sending and receiving) a single bit of data ranges from 15.2 μJ to 18.3 μJ, whereby the exact value depends on the transmit power level. Hence, the comparison of energy cost between the Simplified Kerberos Protocol (SKP) and our Dynamic Authentication and Key Establishment (DAKE) protocol is as follows:

**Table 6.** The comparisons of Energy Cost.

**Protocol**	**Total Length (bits)**	**Req. Energy**

SKP	2,592	39.5∼47.5 mJ
DAKE	1,824	27.7∼33.4 mJ

According to this comparison, our protocol can save about 30% of communication energy.

### 6.2. Eschenauer and Gligor Scheme

One of the popular symmetric key pre-distribution schemes was proposed by Eschenauer and Gligor [5]. This scheme relies on probabilistic key sharing among the nodes of a random graph and uses a simple shared-key discovery protocol for key distribution, revocation and node re-keying.

Prior to Distributed Sensor Network (DSN) deployment, it distributes a ring of keys to sensor nodes, each of which consists of randomly chosen *k* keys from a large pool of *P* keys, which is generated offline. Because of the random choice of keys from the key pool, a shared key may not exist between some pairs of nodes. Although a pair of nodes may not share a key, if a path of nodes sharing keys pair-wise exists between the two nodes at network initialization, the pair of nodes can use that path to exchange a key that establishes a direct link. Therefore, full shared-key connectivity offered by pair-wise private key sharing between every two nodes becomes unnecessary.

**Figure 4.**
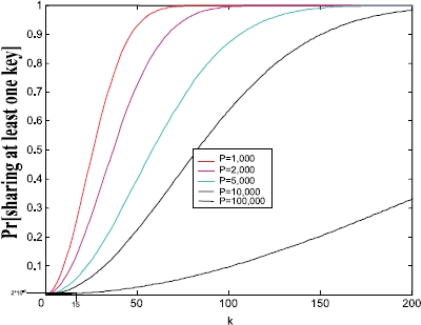
The probability of sharing at least one key.

Figure 4 [5] shows the probability of sharing at least one key when two nodes choose *k* keys from a pool of size *P*. For a pool size *P* = 100,000 keys, 250 keys need to be distributed to any two nodes to have the probability *p* = 0.5 that they share a key in their key ring.

**Figure 5.**
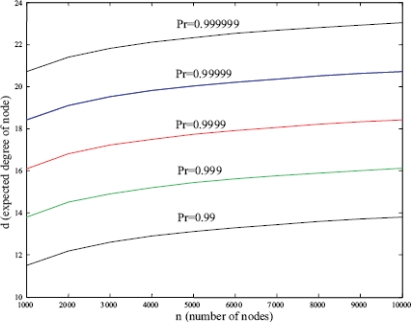
The relationship between expected degree of node and number of nodes.

In order to ensure 99% probability of successful connection, *i.e.*, sharing at least one key between two nodes, a network with 10000 nodes expects almost 14 degrees of node. If 99.999% connection probability is demanded, 20 degrees of node is needed. Figure 5 [5] shows the relationship between expected degree of node and number of nodes.

**Figure 6.**
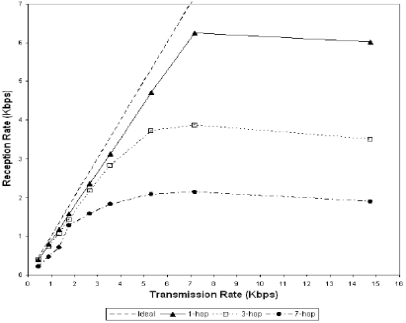
The performance of reception rates.

However, in a practical testing environment [23], the performance drops significantly when the number of hops increases between two ends. Figure 6 [23] shows a real measuring result of reception rate based on different hops. It shows that even for a seven hop network the throughput becomes very small (less than 2 Kbps).

In contrast, thanks to the average number of hops between a sensor and its nearest cluster head is about three, our Dynamic Authentication and Key Establishment protocol with the distribution mode has the higher connective probability (100%) and less memory cost than those pre-distribution schemes without considerable increment of communication.

## 7. Conclusions

In this paper, we have proposed an efficient and scalable protocol to establish and update the authentication key between any pair of sensor nodes in a dynamic wireless sensor network. Our protocol has the following features:

- It is suitable for both static and dynamic WSNs. Any pair of nodes can establish a key for secure communication.
- A roaming node only deals with its closest router for security. There is no need to change the rest of routing path to the base station.
- The base station can manage a revocation list for lost or compromised roaming nodes.
- The system is scalable and resilient against node compromise.

After comparing with some of the popular and latest protocols used in WSNs, our protocol could save about 30% in communication energy, and has the higher probability (100%) of sharing a key between two sensor nodes with less memory cost than those pre-distribution schemes, without incurring in a considerable amount of communication.

## Figures and Tables

**Table 1.** The structure of Key Cache.

**Key Cache in Sensor Node N**		
Correspondence Node ID	Key	Key Lifetime

*BS*	*K_BN_*	*T_BN_*
*node_i_*	*K_Ni_*	*T_Ni_*
… …	… …	… …
*node_j_*	*K_Nj_*	*T_Nj_*
*PreSharedKey_x_*	*K_x_*	*T_x_*
… …	… …	… …
*PreSharedKey_y_*	*K_y_*	*T_y_*

**Table 2.** The initial key entry.

**Correspondence Node ID**	**Key**	**Key Lifetime**

*node_R_*	*R_0_*	*0*

**Table 3.** The structure of Key Table in basestation.

**Key Table in Base Station**		
***Node ID***	***Key Stuff***	***Key Lifetime***

*node_i_*	*K_Bi_*	*T_Bi_*
… …	… …	… …
*node_j_*	*K_Bj_*	*T_Bj_*

**Table 4.** Message length of the simplified kerberos protocol.

**Message**	**Length (bits)**	**Blk.**	**Sub-Total Length**

AS_REQ	160	-	160 + 256
AS_REP	672	6	768 + 256
AP_REQ	448	4	512 + 256
AP_REP	64	1	128 + 256
All messages	1,344	11	2,592

**Table 5.** Message length of our protocol.

**Message**	**Length (bits)**	**Blk.**	**Sub-Total Length**

Req	352	-	352 + 256
Appv	384	3	384 + 256
Notice	320	-	320 + 256
All messages	1,056	11	1,824
